# Special Issue: Porous Ceramics, Glasses and Composites, Volume II

**DOI:** 10.3390/ma16175971

**Published:** 2023-08-31

**Authors:** Francesco Baino

**Affiliations:** Institute of Materials Physics and Engineering, Department of Applied Science and Technology, Politecnico di Torino, Corso Duca degli Abruzzi 24, 10129 Turin, Italy; francesco.baino@polito.it

This Special Issue, titled “Porous Ceramics, Glasses and Composites, Volume II”, aims to present an up-to-date overview of the synthesis/fabrication, characterization, and applications of porous materials, with a special focus on ceramics, glasses, and composites.

There are many examples of natural ceramic-based porous materials that are widely researched and investigated, such as cancellous bone in mammals, which is actually a composite mainly made of hydroxyapatite and collagen that are exquisitely interlocked to form a three-dimensional trabecular architecture [1]. Synthetic porous materials are sometimes designed and fabricated following a bioinspired approach in order to optimize the performance of the product/device [2]. In recent years, the importance of porous materials has significantly grown because of their versatile industrial, high-tech, and popular applications, covering multiple fields from energy exploitation to biomedicine. Nowadays, porous ceramics are used to fabricate a huge variety of devices, such as hot gas or dust collectors, absorbers, thermal insulators, dielectric resonators, engine components for the automotive industry, biomaterials, drug delivery systems, phase separation and filtration devices, etc. The wide use of these materials relies on their appealing properties, such as chemical, biological, and thermal stability/resistance, refractoriness, low thermal conductivity, and relatively low density. If exhibiting open and interconnected pores, porous ceramics allow liquid and gases to flow in and out and, thus, their mass transport properties become of utmost importance [3]. In this regard, permeability confers to these materials the ability to filter, deliver, and release different fluids, substances, and particles. In the fabrication of porous ceramic materials, a key factor is represented by the control of pore characteristics (e.g., amount, size, geometry, interconnectivity, etc.) that can be tailored by properly setting the parameters in several processing methods. Furthermore, the evolution of additive manufacturing technologies and the progressive spreading of these methods, even in industrial contexts at an affordable cost, has opened new horizons in terms of the improved performance of the products, higher control on shape/dimensions, reliability, and ease of scalability [4,5]. An important role is also played by the features of raw materials, type of binder used, and sintering parameters, which can all affect the final porosity in terms of pore volume and pore size distribution.

The research topics covered by this Special include, but are not limited to, the following applications: thermal and acoustic insulation; construction; filtration/separation; catalysis; biomedical field; diffusion in porous media. The use of porous materials in the circular economy, the development of hierarchical porous systems, as well as studies on the modeling of the properties and processes are highly appealing topics. Research papers concerning the structure–property–function relationships in porous ceramics, glasses, and composites are also key to this Special Issue.
